# Airway remodeling in asthma: what really matters

**DOI:** 10.1007/s00441-016-2566-8

**Published:** 2017-02-11

**Authors:** Heinz Fehrenbach, Christina Wagner, Michael Wegmann

**Affiliations:** 1 Division of Experimental Pneumology, Priority Area Asthma & Allergy, Research Center Borstel, Leibniz Center for Medicine and Biosciences, Parkallee 1-40, 23845 Borstel, Germany; 2 Airway Research Center North (ARCN), German Center for Lung Research (DZL), Borstel, Großhansdorf, Kiel, Lübeck, Germany; 3 Junior Research Group of Invertebrate Models, Priority Area Asthma & Allergy, Research Center Borstel, Leibniz Center for Medicine and Biosciences, Parkallee 1-40, 23845 Borstel, Germany; 4 Leibniz-ScienceCampus Evolutionary Medicine of the Lung (EvoLUNG), Kiel, Germany; 5 Junior Research Group of Asthma Mouse Models, Priority Area Asthma & Allergy, Research Center Borstel, Leibniz Center for Medicine and Biosciences, Parkallee 1-40, 23845 Borstel, Germany

**Keywords:** Asthma, Airway remodeling, Airway pathology

## Abstract

Airway remodeling is generally quite broadly defined as any change in composition, distribution, thickness, mass or volume and/or number of structural components observed in the airway wall of patients relative to healthy individuals. However, two types of airway remodeling should be distinguished more clearly: (1) physiological airway remodeling, which encompasses structural changes that occur regularly during normal lung development and growth leading to a normal mature airway wall or as an acute and transient response to injury and/or inflammation, which ultimately results in restoration of a normal airway structures; and (2) pathological airway remodeling, which comprises those structural alterations that occur as a result of either disturbed lung development or as a response to chronic injury and/or inflammation leading to persistently altered airway wall structures and function. This review will address a few major aspects: (1) what are reliable quantitative approaches to assess airway remodeling? (2) Are there any indications supporting the notion that airway remodeling can occur as a primary event, i.e., before any inflammatory process was initiated? (3) What is known about airway remodeling being a secondary event to inflammation? And (4), what can we learn from the different animal models ranging from invertebrate to primate models in the study of airway remodeling? Future studies are required addressing particularly pheno-/endotype-specific aspects of airway remodeling using both endotype-specific animal models and “endotyped” human asthmatics. Hopefully, novel in vivo imaging techniques will be further advanced to allow monitoring development, growth and inflammation of the airways already at a very early stage in life.

## Introduction

With more than 241 million prevalent cases in 2013 (Global Burden of Disease Study 2013 Collaborators 2015), asthma is one of the most common lung diseases worldwide and it is expected that the number of people suffering from this chronic lung disease increases to about 300 million in 2025 (Croisant 2014). In 2013, asthma ranked 15th among all diseases worldwide with regard to years lived with the disability (YLD) over all age groups (Global Burden of Disease Study 2013 Collaborators 2015). Notably, asthma is most prevalent in children with highest YLDs observed in the age group 5–14 years (ranked 6th worldwide, even 2nd in the group of developed countries) (see http://vizhub.healthdata.org/gbd-compare/). On the basis of disability-adjusted life years, the impact of asthma is estimated being similar to other major chronic diseases such as diabetes or Alzheimer disease (Croisant 2014). Even though asthma is generally not seen as a major cause of death, the Global Burden of Disease Study 2013 reported a global age-standardized death rate of 8.0 per 100,000 in 2013, which is equivalent to breast cancer (7.4) or pedestrian road injuries (8.0). Hence, asthma remains among the top 50 causes of global years of life lost (GBD 2013 Mortality and Causes of Death Collaborators 2015).

A brief search for the available literature in PubMed using the strategy “airway AND asthma AND (remodeling OR remodelling)” revealed that, in 1993, the first two papers falling into this category were published and that numbers slowly increased to 203 in 2007. Since then, the number of papers per year are on a relatively constant level of between 200 and 240, which represent only approx. 15% of all publications on “asthma AND airway”. Notably, among these publications, about 50–60 per year are review papers, i.e., 20–40% of all publications. To avoid carrying coals to Newcastle, in the present review we will not try to review everything already well reviewed by others (Elias 2000; Holgate et al. 2000; Saetta and Turato 2001; Jeffery 2004; Hogg 2004; Bai and Knight 2005; Boulet and Sterk 2007; Al-Muhsen et al. 2011; Martinez and Vercelli 2013; Saglani and Lloyd 2015). Instead, we will focus on some aspects related to a methodologically sound quantification as a prerequisite for the reliable assessment of airway remodeling and on what we can learn from invertebrate to primate animal models to better understand the mechanisms underlying physiological versus pathological remodeling.

## Today’s perception of bronchial asthma: the phenotype/endotype concept

The perception of bronchial asthma has fundamentally changed during the last decade. Consequently, the Global Initiative for Asthma (GINA) suggested a new definition of asthma: “Asthma is a heterogeneous disease, usually characterized by chronic airway inflammation. It is defined by the history of respiratory symptoms such as wheeze, shortness of breath, chest tightness and cough that vary over time and in intensity, together with variable expiratory airflow limitation” (Reddel et al. 2015). The definition takes into account that during the last decade asthma turned out to be quite heterogeneous encompassing patients with different phenotypes (i.e., the entity of observable characteristics), which, however, exhibit overlap to variable degrees. Adult asthma was suggested to be not a single disease but a syndrome with one common feature being a variable expiratory airflow limitation (Lötvall et al. 2011; Wenzel 2006). The concept of asthma as a syndrome is also expected to be very helpful in pediatric asthma (Spycher et al. 2010; Lødrup Carlsen and Carlsen 2012). However, the characteristics used for the definition of the various phenotypes are not necessarily directly related to the underlying pathogenetic process(es). Aiming at the development of personalized therapies, i.e., therapeutic approaches targeting key elements of the causative pathomechanism(s), the concept of asthma endotypes was proposed with each endotype being the result of a specific molecular pathomechanism that is distinctly different from the other endotypes (Lötvall et al. 2011; Wenzel 2012; Agache et al. 2012). Until today, probably the (one and only) most clearly defined asthma endotype is the one termed the Th2 (T helper type 2)-high endotype, which is characterized by a T helper lymphocyte type 2-driven inflammation (Fahy 2015). Anti-inflammatory therapies targeting Th2 cytokines such as interleukin (IL)-4, IL-5 and IL-13 have consistently exhibited beneficial effects in adult patients diagnosed as Th2-high asthmatics on the basis of some emerging biomarkers related to the IL-13 response, such as periostin or FeNO (Bhakta and Woodruff 2011; Ingram and Kraft 2012; Fahy 2015; Fajt and Wenzel 2016). Efforts are being undertaken to reveal additional novel biomarkers that may help in identifying and distinguishing further endotypes (Zissler et al. 2016).

The current phenotype/endotype concept of asthma has a strong focus on clinical and inflammatory characteristics and omitted aspects of airway remodeling, one important feature of asthma, as was recently emphasized (Saglani and Lloyd 2015). Today, it is unclear whether the differences in airway remodeling parameter values observed between individual patients define specific remodeling phenotypes and how these may relate to clinical or inflammatory phenotypes or are even linked to a specific endotype.

## Defining airway remodeling in asthma: physiological versus pathological processes

Most review papers define airway remodeling quite broadly as any change in composition, distribution, thickness, mass or volume and/or number of structural components observed in the airway wall of patients relative to the airway wall of normal healthy individuals (Bergeron et al. 2009; Bai 2010; Hirota and Martin 2013). Changes have been described for various tissues in asthma patients, such as airway epithelium (e.g., epithelial shedding, goblet cell hyperplasia, basal membrane thickening), peribronchial interstitial tissue (e.g., subepithelial fibrosis), airway smooth muscle cells (e.g., hyperplasia and/or hypertrophy), nerve tissue (e.g., increased neurite sprouting) and bronchial vasculature (e.g., barrier dysfunction, angiogenesis) (Undem et al. 1999; Beckett et al. 2003; Jeffery 2004; Al-Muhsen et al. 2011). The observation of an inflammatory infiltrate characterized by eosinophilic granulocytes and CD4^+^ Th cells in the airways of (probably Th2-high) asthmatics (Saetta and Turato 2001), sometimes being the result of a longstanding inflammatory process, was suggested as a *conditio sine qua non* of the definition of airway remodeling in asthma (Hirota and Martin 2013). Although airway remodeling has been reported for other chronic lung diseases such as chronic obstructive pulmonary disease (COPD), some structural changes of the airways appear to be distinctly different when comparing asthma and COPD as reviewed recently (Jones et al. 2016). The evidence accumulated until now suggests that airway remodeling is associated with a progressive loss of lung function, a view which still has to be considered a hypothesis because therapies targeting airway remodeling are still missing (Pascual and Peters 2005).

In this review, we adopt the suggestions made by Jeffery (2001, 2004) and distinguish two types of airway remodeling, i.e., physiological remodeling on the one hand and pathological remodeling on the other. *Physiological airway remodeling* comprises those structural changes, which occur regularly during normal lung development and growth leading to a normal mature airway wall or that occur as an acute and transient response to injury and/or inflammation ultimately resulting in restoration of a normal airway structure. Structural alterations that occur as a result of either disturbed lung development or as a response to chronic injury and/or inflammation leading to persistently altered airway wall structures and function are considered as *pathological airway remodeling*.

The most relevant implications of these definitions are:

- Unless quantitative analyses of airway structural characteristics are used, objective evidence of structural deviations from the normal healthy condition cannot be provided without doubt.
- Although airway remodeling is frequently associated with airway inflammation, remodeling cannot be considered being a secondary phenomenon to inflammation in every single case.
- Airway remodeling may be a primary event in asthma pathogenesis if it is the result of disturbed lung development.
- Unless the kinetics of these processes can be revealed, which is very difficult in humans, it will be very difficult to distinguish an acute and transient response from a chronic reaction and, thereby, unequivocally differentiate between physiological and pathological processes. In the absence of data on the kinetics, additional criteria/biomarkers are badly needed and animal models can be very helpful in that they allow for kinetic studies.

Consequently, the following major questions will be addressed:

- what are reliable quantitative approaches to assess airway remodeling,
- are there any indications supporting the notion that airway remodeling can occur as a primary event, i.e., before any inflammatory process was initiated,
- what do we know about airway remodeling being a secondary event to inflammation and
- what can we learn from animal models in the study of airway remodeling for distinguishing physiological and pathological airway remodeling and the underlying, potentially differing pathomechanisms?

## Quantitative approaches to assess airway remodeling

The initial approach to assess airway remodeling, both in humans and in animal models, has been the histologic analysis of two-dimensional sections by means of light, fluorescence or electron microscopy. Recent technological advances allowed the implementation of high-resolution imaging into radiologic analyses of airway morphology (Hartley et al. 2016). These approaches, however, are beyond the expertise of the authors and therefore this review will focus on microscopy-based approaches only.

In 2010, a joint task force of the American Thoracic Society (ATS) and the European Respiratory Society (ERS) published an Official Research Policy Statement paper that critically reviewed the state-of-the-art stereological methods in lung morphometry and defined standards to promote comparability of morphometric studies in pulmonary research (Hsia et al. 2010). This landmark paper is suggested as the starting point for everyone designing new studies of airway remodeling and is a benchmark paper when evaluating published data. As was emphasized by this task force, the quantification of structures is based upon the three-dimensional (3D) physical attributes of its components. When two-dimensional (2D) sections are used for quantitative analysis, only incomplete information about the 3D structure are obtained, which bears a high risk of misinterpretations and false conclusions (Hsia et al. 2010). This risk is particularly prominent for the airway tree, which is highly anisotropic and exhibits marked qualitative and quantitative changes in airway wall structure from proximal to distal airway generations (Crystal et al. 1997; Mauroy et al. 2004). Consequently, quantitative approaches to assess remodeling of airways have to take into account that both the orientation of a 2D section relative to the airway’s longitudinal axis and the location along the airway tract (i.e., airway hierarchy) have marked effects on the quantitative parameters analyzed (Hsia et al. 2010). Therefore, obtaining a collection of an unbiased set of representative tissue samples requires a few but important additional steps during sampling, as described previously for whole lungs obtained from animal models (Hyde et al. 2007; Mühlfeld and Ochs 2013) but also for studying human biopsies (Ferrando et al. 2003; Woodruff and Innes 2006; Bratu et al. 2014).

A few examples could illustrate that using quantitative stereological approaches may challenge some generally accepted views.

### Epithelial cell shedding

**Fig. 1:**
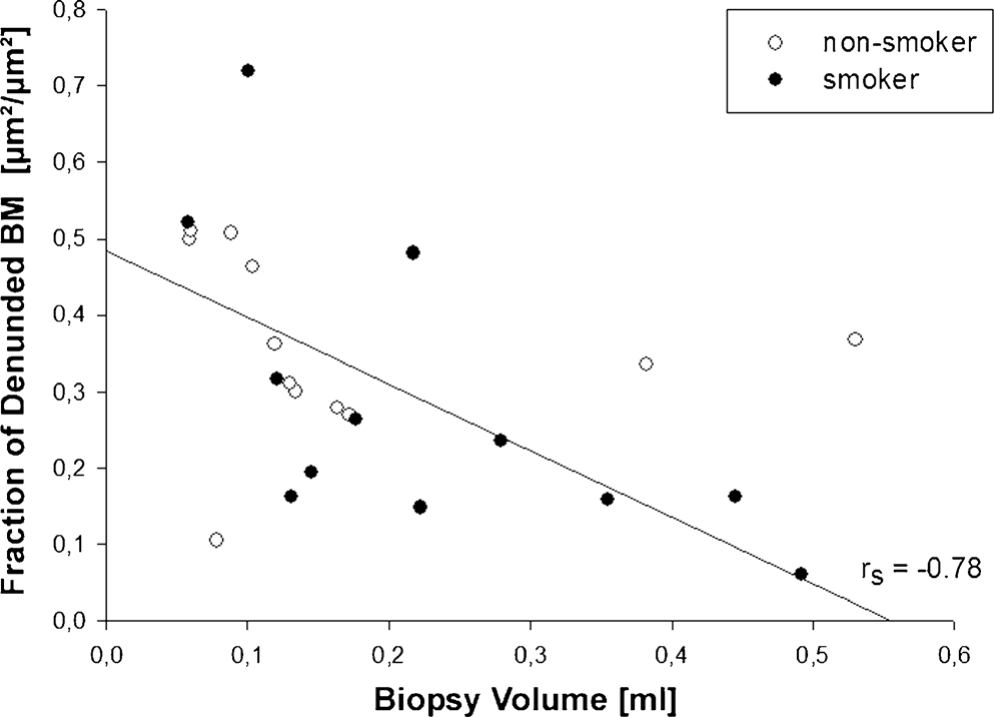
Fraction of epithelial basal membrane (*BM*) of human endobronchial biopsies exhibiting complete denudation is inversely correlated with biopsy volume (=size), which was estimated according to the Cavalieri Principle. Figure by courtesy of Dr. V.A. Bratu, modified from Bratu (2008); Fig. 3.4c

Today, it is widely accepted that the airway epithelium, which is in almost^1^ direct contact to the inhaled air, is far more than just a passive physical barrier to what is inhaled during breathing (Tam et al. 2011). The epithelium exerts various functions that help maintain a healthy lung such as particle clearance, fluid balance, innate immune responses. Direct injury to the airway epithelium induced by various triggers has long been widely accepted as a very early, may be the initial, step in asthma pathogenesis (Al-Muhsen et al. 2011; Hirota and Martin 2013; Holt et al. 2014). This notion is in part based on the qualitative observation of a denuded epithelial basal lamina in biopsies of asthmatics, which was interpreted as the result of epithelial cell shedding and a histological reflection of the loss of airway epithelial function (for references of original studies, see, e.g., Bergeron et al. 2009; Fajt and Wenzel 2016). A computer-based quantitative study of bronchial biopsies obtained from 14 mild and moderate human asthmatics, however, revealed that there were no differences in the degree of epithelial desquamation in comparison to biopsies from 12 healthy subjects (Ordoñez et al. 2000). Using glycol methacrylate as embedding medium for the biopsies, a section thickness of 2 μm could be achieved, which allowed an excellent presentation of all structures in the microscopic images. The authors suggested that epithelial desquamation in endobronchial biopsies in asthmatics is an artifact of tissue sampling and not a true pathologic feature of asthma. Although this was controversially discussed (Holgate et al. 2001), our own data demonstrate that the degree of epithelial desquamation in human endobronchial biopsies increases with decreasing biopsy size (Fig. 1). The smaller the biopsy, the more mechanical forces may affect the tissues during collection and embedding, which supports the notion that epithelial desquamation is highly prone to artefactual damage. Therefore, epithelial shedding is a questionable phenotypic characteristic of asthma. Without any doubt, however, it is very well supported that dysregulated airway epithelial cell functions are a central element in the pathogenesis of asthma (Holgate 2007, 2011a, b; Fahy and Locksley 2011).

### Epithelial basement membrane thickening

The basement membrane or basement membrane zone of the airways is an extracellular structure specialized for the attachment of the epithelium to the underlying extracellular matrix (Evans et al. 2010). In the electron microscope, three layers can be distinguished: the lamina lucida, the lamina densa and the lamina reticularis, which have been shown to also differ in their chemical composition. The lamina reticularis, the basal portion of the basement membrane, can also be seen with the light microscope and is also referred to as reticular basement membrane (RBM) or subepithelial basement membrane. RBM thickening was suggested to be pathognomonic of asthma (Jeffery 2004). However, it has been reported to also occur in children with cystic fibrosis (Hilliard et al. 2007). Moreover, RBM thickening was also observed in adult COPD patients with RBM thickness being not significantly different from adult subjects with asthma (Liesker et al. 2009). When analyzing RBM thickening in the airways, again one has to take into account that there is marked variation along the airway tree, with the RBM becoming thinner as it extends from the trachea into the small airways (Evans et al. 2010). In addition, RBM thickness as represented in microscopic sections strongly depends on how much the sectioning angle deviates from the ideal situation of a section normal to the RBM surface. Therefore, exclusion of obliquely to tangentially cut tissue is a step regularly implemented into the measurement of RBM thickness, although using largely subjective criteria. Implementation of design-based stereological protocols is rare, although this is the only way to guarantee random tissue orientation (i.e., isotropic, uniformly random orientation) ensuring that tangential cuts occur with a known distribution and can be handled systematically (Ferrando et al. 2003; Hsia et al. 2010). Ferrando et al. (2003) made comparisons using two classical procedures in parallel. Although RBM was significantly thicker in asthmatics than in healthy subjects, the measurements made by using design-based stereology were approximately 30% smaller than measurements made with the two classical procedures. Notably, the mean coefficient of error for repeat measurements (i.e., the reproducibility of the measurements) was 0.06 for the stereological approach, which is by far preferable compared with the 0.19–0.30 in the other approaches (Ferrando et al. 2003). Implementation of such an unbiased procedure as an objective correction for tangential cuts could be an important step for standardization of protocols and thus would help ensure better comparisons of measurement data across studies and laboratories. Accepting that reliable methods for the quantitative assessment of RBM thickening are crucial for obtaining sound data, reports on therapies successfully reducing RBM thickness in asthma patients, reviewed recently by Durrani et al. (2011), should be critically re-evaluated.

### Smooth muscle hyperplasia and hypertrophy

The potential role(s) of airway smooth muscle cells in the pathogenesis of asthma symptoms, in particular with regard to airway hyperresponsiveness, has been comprehensively reviewed by others (An et al. 2007; Gosens and Grainge 2015). It is widely accepted that the total amount of smooth muscle is increased in asthma (Jeffery 2004; Fixman et al. 2007; Durrani et al. 2011). The increase seems to involve both small and large airways (James et al. 2012) and may be related to the clinical severity and duration of asthma (Bai et al. 2000). In principle, the increase in muscle mass can be achieved by an increase in cell number (hyperplasia), by increase in cell volume (hypertrophy), or a combination of both and by additional immigration of myofibroblasts (Bara et al. 2010). Distinctly different molecular mechanisms may be considered leading to hyperplasia or hypertrophy and both may be associated with distinct functional consequences. Conclusive demonstration of hyperplasia, i.e., increase in cell number, is only feasible if an unbiased design-based stereology approach such as the physical or optical disector is applied (Sterio 1984; Hsia et al. 2010; Gruber et al. 2012). Using an assumption-based quantitative approach and the mean cell diameter across the nucleus as surrogate for cell size, hypertrophy was reported to already be present in mild-to-moderate asthmatics and being even more pronounced in severe asthmatics (Benayoun et al. 2003). However, using design-based stereology, a biopsy study in adult patients with mild-to-moderate asthma demonstrated hyperplasia of smooth muscle cells, i.e., an increase in cell number per volume of tissue, whereas no significant increase in cell size, i.e., mean cell volume in μm^3^, was seen (Woodruff et al. 2004). Notably, Woodruff et al. (2004) did not find any differences in gene expression of smooth muscle cells isolated by laser-capture microdissection from bronchial biopsies of asthmatics and healthy controls with regard to the cellular phenotype. Regamey et al. (2008) demonstrated by design-based stereology that both hyperplasia and hypertrophy occur in children with mild-to-severe asthma but that hyperplasia was the most prominent contributor to the increase in smooth muscle mass. In this study, smooth muscle cell hyperplasia revealed to be not limited to children with asthma but to also be present in cystic fibrosis and non-cystic fibrosis bronchiectasis. Differentiation of hyperplasia versus hypertrophy is important when considering airway smooth muscle as a potential therapeutic target. Exciting new data suggest that bronchoconstriction as a result of airway smooth muscle contraction appears sufficient to induce airway remodeling via processes triggered by mechanical forces and independent of the inflammatory response (for review see Grainge et al. 2011; Gosens and Grainge 2015). These studies indicate that airway smooth muscle cells may not simply be secondary effector cells responding to an already ongoing pathogenic process but in contrast may also be at the forefront of disease initiation.

## Airway remodeling as a secondary event to inflammation

In fact, airway remodeling in allergic bronchial asthma is discussed to be the result of a chronic inflammatory response entailing on the one hand permanent airway tissue destruction and on the other hand chronic tissue repair. Thus, chronic airway inflammation can be described as the major force driving the processes leading to most aspects of airway remodeling. This “inflammation theory” is mainly supported by the finding that steroid treatment in asthmatic patients does not only reduce airway inflammation but also has beneficial effects on airway remodeling (Trigg et al. 1994; Olivieri et al. 1997; Laitinen et al. 1997; Hoshino et al. 1998, 1999, 2001; Sont et al. 1999; Ward et al. 2002; Chetta et al. 2003). Infiltrating cells like T helper (Th) cells, eosinophils, neutrophils and mast cells interact with resident cells of the airways such as fibroblasts, smooth muscle cells, neuronal cells, epithelial cells and endothelial cells by the release of a plethora of cytokines, enzymes, metabolites and growth factors creating a signaling environment that—under chronic conditions—results in airway remodeling.

T helper cells—in allergic bronchial especially Th2 cells—orchestrate the allergic inflammatory response by releasing a characteristic array of cytokines including IL-4, IL-5, IL-9 and IL-13. Each of these cytokines has prominent functions in directing the production of allergen-specific IgE, recruitment of eosinophils or development of AHR; however, whether they directly have an impact on airway remodeling is still a matter of debate. As already mentioned, gene-targeted mouse strains overexpressing the cytokines IL-4, IL-5, IL-9, IL-11, or IL-13 spontaneously develop airway inflammation, AHR, mucus hyperproduction and airway remodeling (Rankin et al. 1996; Tang et al. 1996; Lee et al. 1997; Temann et al. 1998; Zhu et al. 1999). After these initial studies, further experiments provided deeper insight into the contribution of each of these Th2-type cytokines to airway remodeling. At least the effects of IL-4, IL-5 and IL-9 are either dependent on IL-13 or promote airway remodeling by supporting the infiltration and activation of further inflammatory cells like eosinophils and mast cells (Cohn et al. 1997; Temann et al. 2002; Justice et al. 2002; Whittaker et al. 2002). IL-5 is essential for terminal differentiation, maturation, migration and survival of eosinophils in peripheral tissues but has no direct effect on airway remodeling in mice (Walter et al. 2001). IL-9 enhances mucus production in an IL-13-dependent manner (Steenwinckel et al. 2007) but also influences accumulation of mast cells in asthmatic airways (Temann et al. 2002). The only Th2-type cytokine that has been identified to have profound effects on airway structural cells is IL-13: in mice, it induces mucin expression and mucus metaplasia in both airway epithelial cells and submucosal glands through activation of STAT-6 and also plays a key role in goblet cell hyper/metaplasia in humans (Kuperman et al. 2002; Atherton et al. 2003). Furthermore, IL-13 induces the release of the profibrotic TGF-β by epithelial cells (Richter et al. 2001; Malavia et al. 2008).

Th17 cells are characterized by the secretion of IL-17 and the expression of the transcription factor RORc in humans or RORγt in mice, respectively. By releasing several proinflammatory cytokines, they can amplify inflammatory responses: e.g., through release of IL-17 they are able to induce IL-8 secretion in epithelial cells, which in turn leads to neutrophil recruitment. Though they are not the only source of IL-17 production in asthmatic patients, their percentage in PBMCs and IL-17 levels in plasma concentrations correlate with disease severity, so that they could play a role especially in severe or corticoid-refractory forms of asthma (Al-Ramli et al. 2009; Takeda et al. 2009; Zhao et al. 2010; Doe et al. 2010). Whether Th17 cells directly contribute to airway remodeling is part of an ongoing discussion. In a mouse model of chronic experimental asthma, the absence of Th17 cells resulted in diminished airway remodeling as demonstrated by reduced staining of collagen fibers and α-smooth muscle actin, although allergic airway inflammation remained unaltered (Zhao et al. 2013). Lu et al. (2015) also used a mouse model of chronic experimental asthma and correlated progressively increasing levels of Th17 cells and IL-17A with peribronchial microvessel density. Neutralization of IL-17 abrogated these signs of vascular remodeling. However, in both studies, lack or reduction of IL-17 as produced by Th17 cells was paralleled by a reduction of inflammatory cell infiltration, neutrophils in the study of Zhao et al. (2013) or eosinophils in the study of Lu et al. (2015), so that it is still not clear whether they indeed have a direct effect on airway remodeling or whether they contribute to this by enhancing the local inflammatory response.

Therefore, most of the effects of T helper cells on airway remodeling appeared to be indirect and to depend on their proinflammatory effect promoting the infiltration, especially of eosinophils and mast cells. Under the control of IL-3, IL-5, GM-CSF and eotaxins, eosinophils are recruited to the lung and represent a typical characteristic hallmark of allergic airway inflammation in asthma (Robinson et al. 1999; Sehmi et al. 2003). Their genuine function in the immune system has been described as major effector cells in defense against invading parasites. Therefore, eosinophils are capable of producing and releasing a bunch of tissue-damaging cationic proteins, enzymes and reactive oxygen species as well as of cytokines, chemokines, cysteinyl leukotrienes and eicosanoids (Kariyawasam and Robinson 2006). By releasing these products, eosinophils provoke airway remodeling by two different ways, on the one hand these cells appear to be the major source of tissue damage during allergic airway inflammation and on the other hand they stimulate tissue repair processes. Cationic eosinophil-derived granule proteins (EDGPs) play a pivotal role in the first type of process since they are toxic to cells and human tissues, as demonstrated by studies using eosinophil- or EDGP-deficient mouse strains (Denzler et al. 2000; Lee et al. 2004b; Specht et al. 2006; Doyle et al. 2013; Jacobsen et al. 2014). They comprise eosinophil cationic protein (ECP), eosinophil-derived neurotoxin (EDN), eosinophil peroxidase (EPX), galectin 10 (Gal-10) and major basic proteins (MBP-1 and -2) (Acharya and Ackerman 2014). In vitro experiments demonstrated that MBP-1, which can disrupt the lipid bilayer membrane of the cell in vitro (Gleich et al. 1993), EPX that produces hydrogen peroxide and EDN, a member of the ribonuclease A superfamily, are highly cytotoxic and, thus, could play a role in epithelial shedding as observed in allergic bronchial asthma. Like EPX and MBP-1 ECP is a highly cationic polypeptide with marked toxicity and neurotoxicity (Fredens et al. 1982) and has been implicated into the development of AHR in asthmatic patients. Besides the release of these cytotoxic EDGPs, eosinophils contribute to aggravation of the local inflammatory response by producing proinflammatory mediators such as leukotrienes. While these eosinophil products lead to tissue destruction and promote airway inflammation, eosinophils are also the main source of TGF-β, a cytokine with anti-inflammatory as well as profibrotic properties. Hence, using an anti-IL-5 antibody, which depleted eosinophils in asthmatic patients, the levels of TGF-β in the lung, TGF-β expression in eosinophils and signs of airway remodeling were reduced in parallel (Flood-Page et al. 2003). Accordingly, in a mouse model of experimental allergic asthma, corticosteroid treatment not only diminished eosinophil numbers in the airways but also TGF-β expression in the lung and airway remodeling, as assessed by myofibroblast accumulation and peribronchial fibrosis (Miller et al. 2006). These effects can be explained by the multifaceted effects of TGF-β on structural cells of the airway wall. Thus, it induces peribronchial fibrosis by stimulating fibroblasts to produce collagen and fibronectin. Furthermore, TGF-β dampens the production of enzymes like collagenase that degrade extra-cellular matrix proteins and increases the production of the respective enzyme inhibitors called TIMPs (tissue inhibitor of metalloproteases) (Wynn 2007). Additionally, TGF-β contributes to smooth muscle hyperplasia by stimulating smooth muscle cell hyperplasia (Xie et al. 2007), by promoting the differentiation of fibroblasts into myofibroblasts (Michalik et al. 2009) and by enhancing the migration of smooth muscle cells towards the epithelium to form new muscle bundles (Ito et al. 2009).

Whether neutrophils contribute to airway remodeling like eosinophils is not that clear, which could be because increased neutrophil numbers are not a common feature of asthmatic patients. These cells are found in elevated numbers in severe and corticosteroid-refractant forms of asthma that have also been described as “neutrophilic” asthma (Jatakanon et al. 1999; Foley and Hamid 2007), or in patients that suffer from acute asthma exacerbations (Bhakta and Woodruff 2011). In such patients, neutrophil infiltration correlated with excessive mucus plugging, mucus gland hypertrophy, persistent airflow limitation, shedding of the airway epithelium and thickening of the basement membrane suggests that neutrophils are indeed involved in processes leading to airway remodling (Pepe et al. 2005; Shaw et al. 2007). As the typical effector cell population in anti-microbial Th1-type immune responses, neutrophils are capable of releasing cytokines and growth factors as well as enzymes and metabolites targeting invading pathogens. Among these factors are IL-9, TGF-α and TGF-β, which have have potent effects on mucus production and subepithelial fibrosis as described above (Foley and Hamid 2007). The most prominent enzymes released by neutrophils are matrix metallo protease 9 (MMP-9) and human neutrophil elastase. MMP-9 exhibits elastinolytic and gelatinolytic activities and its levels in the lung have been shown to correlate with asthma severity as well as with the degree of subepithelial fibrosis in patients suffering from asthma (Wenzel et al. 2003; Cundall et al. 2003). Like HNE, its proteolytic activity could possibly contribute to tissue damage and, thus, to airway remodeling. HNE is capable of degrading collagen and elastin fibers, which impairs the integrity of the airway wall under pathologic conditions. Furthermore, HNE triggers secretion of TGF-β by airway smooth muscle cells via an NF-κB-dependent pathway, which could additionally amplify repair processes and, thus, remodeling of the airway wall (Lee et al. 2006).

Mast cells can be described as the first responders to allergen contact in local allergic responses. Cross-linking of allergen-specific IgE that is bound to the surface of mast cells via the high-affinity FcεRI leads to release of preformed and de novo synthesis of numerous mediators such as histamine, leukotrienes, prostaglandins, cytokines, growth factors, chemokines and enzymes. Within minutes, this results in broncho-constriction, edema and mucus secretion, which is described as the allergic early phase response (Bradding et al. 2006). Although mast cells are more or less resident cells localized at the epithelial barriers of the body, they have been found to migrate into the airway smooth muscle layer and into submucosal glands during asthma pathogenesis. In patients who died from fatal asthma, the numbers of degranulated mast cells positively correlated with a greater degree of smooth muscle shortening and larger submucosal gland area, indicating that mast cells indeed have a profound effect on structural cells of the airway wall (Chen et al. 2004). The majority of such mast cells produce Th2-type cytokines like IL-4 and IL-13 (Brightling et al. 2003). Furthermore, mast cells have been found to act on fibroblasts, myofibroblasts and smooth muscle cells by producing and secreting TGF-β, platelet-derived growth factor (PDGF) and fibroblast growth factor (FGF_2_) (Hirst et al. 1996; Cohen et al. 1997; Kanbe et al. 1999; Cohen et al. 2000; Hashimoto et al. 2001). Another member of the growth factor family is amphiregulin, which is also released by mast cells after FcεRI cross-linking in vitro and its expression is increased in asthmatic patients. Since, in epithelial cell lines, amphiregulin up-regulates mucine gene expression, while in asthma patients its expression correlates with the extent of goblet cell metaplasia, mast cells could contribute considerably to mucus hypersecretion in asthma (Okumura et al. 2005). Furthermore, mast cell-derived amphiregulin also induces proliferation of human primary lung fibroblasts, so that mast cells could also play a role in the development of subepithelial fibrosis (Wang et al. 2005). Besides these cytokines and growth factors, mast cells are the major source of chymase and tryptase, two enzymes that have profound effects on structural cells of the airway wall. Thus, tryptase has been shown not only to promote collagen synthesis in fibroblasts but also to stimulate proliferation of these cells, as well as of smooth muscle cells, epithelial cells and endothelial cells (Cairns and Walls 1997; Compton et al. 1998; Berger et al. 2001). In contrast, chymase reduces smooth muscle cells proliferation as induced by epidermal growth factor (EGF) and degrades smooth muscle cell pericellular matrix (Tchougounova et al. 2001; Lazaar et al. 2002).

## Airway remodeling as a primary event

In recent years, evidence has accumulated that early life (pre- and postnatal) exposures to various stressors increase the risk of children to develop deficits in lung function, which in the long run may lead to chronic respiratory diseases such as bronchial asthma (Maritz et al. 2005; Krauss-Etschmann et al. 2012). These early origins of lung function deficits have been linked to adverse effects on lung development (Dezateux and Stocks 1997; Martinez 2009; Stocks et al. 2013). Recent studies indicate that maternal smoking during pregnancy and even paternal smoking prior to conception may increase the risk of children-to-be to develop wheezing or asthma later in life (Burke et al. 2012; Perret et al. 2016; Svanes et al. 2016). Conceptually, such prenatal exposures may interfere either directly with morphogenetic processes during lung development (Harding and Maritz 2012) or indirectly via an altered immune state of the mother (Prins et al. 2012; Arck and Hecher 2013). As a (direct or indirect) consequence, changes of the delicately balanced airway geometry (Mauroy et al. 2004) may ensue resulting in relevant alterations of airway function. There is good epidemiological evidence that an inappropriate course of lung growth is associated with early deficits in lung function and the risk to develop asthma in childhood or later in life (Bisgaard et al. 2012; Sonnenschein-van der Voort et al. 2014, 2015). Based on the non-invasive high-speed interrupter technique, which allows for the measurement of high-frequency respiratory impedance in very young children in vivo, flow limitation in preterm infants was suggested to be determined not only by airway diameter and airway obstruction but also by the mechanical properties of the airway walls (Henschen et al. 2006).

Because asthma frequently starts in childhood but unequivocal asthma diagnosis in pre-school children is very difficult, only limited data are available that allow discussing the question whether airway remodeling may be the primary event initiating disease pathogenesis. Therefore, this question cannot be answered satisfactorily today. A few quantitative studies, although not following a design-based stereological approach, addressed the initial occurrence of airway wall remodeling in young to very young children. Pohunek et al. (2005) presented one of the first studies to provide evidence for airway remodeling very early in childhood. In a bronchial biopsy study of 27 children aged 1.2 to 11.7 years, bronchoscoped because of recurrent or chronic respiratory symptoms and re-evaluated 22–80 months later, the thickness of the subepithelial lamina reticularis was observed to be greater in children with bronchial asthma diagnosed at follow-up, compared with children who did not progress to asthma. This suggests that remodeling may be present even before asthma becomes symptomatic. However, remodeling was accompanied by eosinophilic inflammation. The link of eosinophilic inflammation and remodeling (recently reviewed in detail; Saglani and Lloyd 2015), already at the initiation of the disease is indirectly supported by Saglani et al. (2007) who did not find an increase in basement membrane nor in eosinophilic infiltration in bronchial biopsies of children (aged 3.4–25.9 months) with reversible airflow obstruction who, however, presented with severe, prolonged, or atypical symptoms (Pelkonen et al. 2005). Notably, in this study, older children (aged 6–16 years) with difficult asthma had increased basement membrane thickness similar to adult asthmatics. In contrast, an increase in basement membrane thickness accompanied by differences in extracellular matrix components was seen in very young children (aged 4–44 months) with a high risk of developing asthma (presence of atopic eczema or asthmatic mother) compared to a control group (aged 3–38 months) not exhibiting these risk factors (Berankova et al. 2014). The increase was independent of previous wheezing episodes and none of the children had a positive test for food or inhalation allergen sensitization. Data on inflammatory infiltrates were, however, not included in this study. In pre-school children aged 7–57 months, both basement membrane thickening and eosinophilic infiltration of the airway mucosa where observed in confirmed wheezers versus control subjects who had no history of wheezing or lower respiratory symptoms (Saglani et al. 2007). In contrast, a biopsy study in pre-school children with severe wheezing reported a number of characteristics of airway remodeling such as increased basement membrane thickness, increase in airway smooth muscle, vascularity and mucus gland area but failed to detect any relationship with inflammatory cell counts (Lezmi et al. 2015). In a study of 50 children at 3 years of age who underwent lung function measurements and bronchoscopy for recurrent lower respiratory symptoms at a mean age of 1 year, basement membrane thickness and numbers of mucosal mast cells but not eosinophils, at age 1 correlated significantly with the amount of inhalative corticosteroids purchased at 3 years of age (Malmström et al. 2011). However, follow-up data collected from the same children at age 8, did not support an association between infant basement membrane thickness and current asthma symptoms, lung function, or airway responsiveness (Malmström et al. 2015). The authors did not find any difference in infant airway smooth muscle area fraction or basement membrane thickness between the patients with current or past asthma or with no asthma, suggesting that these early life structural changes of the airway wall is not pathognomonic. However, one has to take into account that the quantitative data were not obtained according to unbiased design-based stereology.

Indirect evidence supporting the notion that airway remodeling may be a primary event, at least in some patients, comes from non- or minimally-invasive functional studies that suggested airway wall compliance in vivo being different in infants suffering from wheezing disorders, even when they were asymptomatic (Frey et al. 2000). This study, however, was not designed to distinguish whether the alterations were acquired (e.g., by inflammatory processes) or whether they pre-existed at the onset of wheezing. Non-invasive measurement of exhaled nitric oxide fraction (FeNO), a marker of pulmonary inflammation, was assessed in a prospective birth cohort to investigate if FeNO levels after birth in unselected newborns were associated with asthma or atopy at school age (Usemann et al. 2016). This prospective study demonstrated that postnatal FeNO measured in unselected healthy newborns was not associated with asthma diagnosis at school age and the authors speculated that NO metabolism might play a role in the pathophysiology of childhood asthma and atopy only after exposure to environmental factors at pre-school age. Since indicators of airway wall structure were reported in other studies to be already altered very early in life (see references above), one may hypothesize that airway remodeling can indeed precede airway wall inflammation and thus may present as a primary event. Deciphering how early-life or even prenatal risk factor exposures may be mechanistically linked to airway structural and functional changes later in life will be both highly interesting from a scientific perspective as well as highly important for understanding and preventing the initiation not only of bronchial asthma but of chronic respiratory diseases in general (Stocks et al. 2013). Identification of relevant mechanisms will help us in the development of effective strategies for the early prevention of such widespread pulmonary diseases. A lot of work awaits to be done.

## Animal models in the study of airway remodeling

### Mammals

#### Mouse

Since the 1990s, mouse models of experimental allergic asthma have been utilized extensively to investigate the pathogenetic mechanisms underlying the formation of the disease and have provided in-depth understanding of the role of various cells and their products in asthma pathogenesis. Over time, a plethora of models has been developed employing different sensitization protocols to the model allergen ovalbumin (OVA) or to clinically more relevant allergens such as house dust mite (HDM), ragweed or cockroach. Once the animal has been sensitized and develop an adaptive Th2 cell-dominated immune response, allergic airway inflammation is directed to the airways by short-term allergen aerosol inhalation or local application of an allergen containing solution. Such short-term models include allergic airway inflammation with infiltration of eosinophils and Th2 cells, increased mucus production and airway hyperresponsiveness (AHR) and thus display key hallmarks of human allergic asthma. However, these models are limited by their short-term nature and, thus, fail to reflect signs of chronicity like chronic airway inflammation, intra-epithelial eosinophils and airway remodeling.

**Fig. 2:**
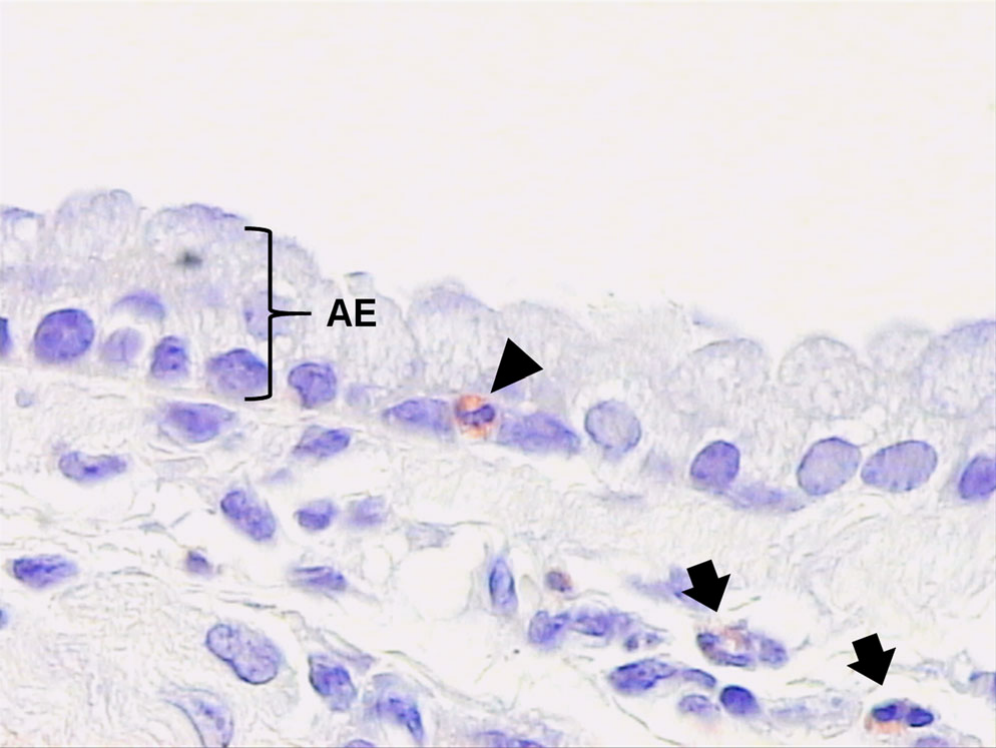
Intra-epithelial eosinophilic granulocyte (arrowhead) in main bronchus of a murine lung chronically challenged with ovalbumin according to the protocol of Wegmann et al. (2005). Tissue was fixed with 4% paraformaldehyde, embedded into glycol methacrylate and the section was Congo Red-stained for eosinophils. *AE* airway epithelium. *Black arrows* indicate eosinophilic granulocytes in subepithelial interstitial tissue

These limitations have been conquered by the development in the 2000s of mouse models of chronic experimental asthma. More or less independently from the mode of sensitization to the allergen, prolongation of allergen challenge was utilized to perpetuate allergic airway inflammation and, thus, the process of airway tissue destruction and repair, ultimately leading to structural changes of the airway wall. We exposed OVA-sensitized Balb/c mice (*Mus musculus*) to an OVA-aerosol for 20 min twice a week for 12 weeks and recorded a change of the inflammatory pattern in the airways as well as signs of airway remodeling (Wegmann et al. 2005). Compared to OVA-sensitized mice exposed only twice to an OVA-aerosol, long-term exposed animals displayed allergic airway inflammation not only of proximal airway sections but also of the entire airway tree. While eosinophils dominated the inflammatory infiltrate in the short-term model, we found no eosinophils infiltrating the airway epithelium. In contrast, intra-epithelial eosinophils were present after long-term OVA aerosol challenge (Fig. 2), although lymphocyte numbers in the airway tissue were much higher than eosinophil counts. A considerable part of these lymphocytes were B cells secreting OVA-specific IgE, IgG1 and IgA, which were nearly absent in the short-term model (Luger et al. 2009). The survival of these cells critically depended on the local production of neurotrophins such as nerve growth factor (NGF) and neurotrophin 3 (NT-3), which were largely produced in the lungs of chronically OVA-challenged animals (Abram et al. 2009). Even after 6 weeks without any further OVA aerosol challenge, lymphocytes were still present in the broncho-alveolar lavage (BAL) fluid and the airway wall, while eosinophil counts in both compartments decreased dramatically over time, although small numbers were still present in the airway tissue (Wegmann et al. 2005). This change in the inflammatory pattern was further associated with markedly increased levels of transforming growth factor β (TGF-β) in the BAL fluid. Accordingly, we found increased collagen fiber deposition, especially in the lamina propria and elevated numbers of adventitial fibroblasts, indicating subepithelial fibrosis. Staining against α-smooth muscle actin further revealed enhanced numbers of myofibroblasts and thickening of the smooth muscle layer, suggesting smooth muscle hyperplasia (Wegmann et al. 2007). Diminishing allergic airway inflammation in this long-term model, either by inhibiting eosinophil chemotaxis inhibitor or by Th2 cell activity, also resulted in reduced goblet cell hyperplasia, smooth muscle cell hyperplasia and subepithelial fibrosis, suggesting that airway remodeling is indeed dependent on an ongoing inflammatory process in the airways (Wegmann et al. 2007; Sel et al. 2008).

Comparable results were achieved by Kumar et al. (2000), who avoided the common problem of tolerance induction and lung parenchymal inflammation after chronic allergen exposure in mice by inhalation of carefully controlled mass concentration of aerosolized OVA. Thus, OVA-sensitized Balb/c mice were challenged with 10–20 mg/m^3^ aerosolized OVA 3 days a week for 8 weeks. Their phenotype of experimental chronic asthma was characterized by chronic airway inflammation, intra-epithelial eosinophils, a local Th2-biased humoral immune response, AHR and signs of airway remodeling, such as subepithelial fibrosis, goblet cell hyperplasia and hypertrophy of the airway epithelium (Temelkovski et al. 1998; Foster et al. 2000, 2002; Kumar et al. 2000). A considerable number of other models have been established in mice that use long-term allergen provocation to induce an acute-to-chronic inflammatory response in the airways to cause abnormal airway injury and to induce repair responses, which ultimately result in airway remodeling. Beneath the above-mentioned structural changes of the airway wall, like subepithelial fibrosis, smooth muscle hypertrophy and goblet cell hyperplasia, these models also display microvascular remodeling and neovascularization. Alterations of the airway epithelium also involve disruption of tight junctions and increased apoptosis of epithelial cells, which result in epithelial shedding and a compromised barrier function of the airway epithelium (Hogaboam et al. 2000; Trifilieff et al. 2000; Dorscheid et al. 2003; Jain et al. 2003; Jungsuwadee et al. 2004).

It is interesting to note that the phenotype of these chronic asthma mouse models can be induced regardless from the allergen (OVA, HDM, *Aspergillus fumigatus*, etc.) or a sensitization protocol that has been used to indicate that once the animal has been sensitized structural changes of the lung are induced by perseverative allergen provocation (Johnson et al. 2004). Since different aspects of sustained airway remodeling persisted even after discontinuation of allergen challenge (Leigh et al. 2002; Cho et al. 2004; Johnson et al. 2004; McMillan and Lloyd 2004; Kumar et al. 2004; Wegmann et al. 2005), these studies demonstrated that mice are indeed capable to mimic allergen-induced responses of the epithelial–mesenchymal trophic unit, leading to various signs of airway remodeling attributed to human bronchial asthma. Therefore, studying the chronic phases of asthma is clearly possible in mice. Furthermore, since such studies revealed that airway remodeling persisted after cessation of allergen challenges well beyond the resolution of allergic inflammation, this suggests that mechanisms independent of inflammation are active in sustaining airway remodeling even when remodeling evolved as a result of allergic airway inflammation (Foster et al. 2000; Leigh et al. 2004; Kumar et al. 2004). The dissociation of airway remodeling and allergic lung inflammation has additionally been supported by findings in transgenic mice as well as in studies using a therapeutic setting that reported beneficial effects on inflammatory processes whereas features of airway remodeling were not affected (Kerzel et al. 2009, 2011; Gregory et al. 2010; Gabehart et al. 2013).

A considerable advantage of asthma mouse models is the availability of gene-targeted or transgenic animals and of a plethora of specific reagents that allow the determination of the particular contribution of individual factors (e.g., mediators, cells) to asthma pathogenesis. Consequently, the role of Th2-type cytokines has been investigated in detail by subjecting genetically modified animals to the protocol for the induction of chronic experimental asthma. Thus, allergen-sensitized animals lacking either IL-5 or IL-13 developed significantly less airway remodeling compared to wild-type animals after chronic allergen challenge (Kumar et al. 2002; Cho et al. 2004). Accordingly, animals overexpressing either IL-5 or IL-13 spontaneously develop airway eosinophilia, AHR and airway remodeling (Lee et al. 1997; Zhu et al. 1999). Comparable phenotypes could be observed in mice overexpressing other Th2-type cytokines such as IL-9 and IL-11 (Tang et al. 1996; Temann et al. 1998). Since all these transgenic animals also spontaneously developed an inflammatory response in the airways, the direct contribution of these cytokines to airway remodeling is still a matter of debate.

The overexpression of classic profibrotic growth factors such as TGF-β and vascular-endothelial growth factor (VEGF) in the lung also produced marked airway remodeling. Hence, conditional overexpression of TFG-β in airway epithelium using a tetracycline-controlled suppressor and reverse tetracycline transactivator system led to profound inflammation of the airways associated with increased collagen deposition, enhanced numbers of myofibroblasts and myocytes in the airway wall and elevated apoptosis of epithelial cells. Lung-targeted VEGF transgenic mice showed an asthma-like phenotype characterized by airway inflammation and AHR together with goblet cell hyperplasia, edema, smooth muscle cell hyperplasia and parenchymal and vascular remodeling (Lee et al. 2004a).

Although the use of mouse models of chronic experimental asthma has provided deep insights into the role of various receptors, cytokines and other factors in the pathophysiology of allergic bronchial asthma and also in the processes ultimately leading to airway remodeling, none of these models has been able to entirely reflect all aspects of the human disease and they have considerable limitations. One critical point is of course the anatomy of the murine lung that differs in a number of aspects from the human organ. The size of the largest intrapulmonary airways of the murine lung is approximately at the level of small airways in humans, so that their physiology as well as the structure of their airway wall display major differences compared to human airways (Hyde et al. 2006). For example, murine airways do not display submucosal glands, so that mucus production is entirely managed by goblet cells and so remodeling processes concerning such glands cannot be investigated (Borthwick et al. 1999). Furthermore, the murine lung lacks a normal systemic circulation to the intra-parenchymal airways, so that processes of neo-vascularization and vessel remodeling of the airway wall are not fully reflected by murine models (Verloop 1949). Mice further display a bronchial branching pattern that is different from humans. In humans, branching is symmetrical and dichotomous with up to 23 generations, while in mice—as in all non-primates—is monopodial and reaches six to eight generations (Gomes and Bates 2002). Another critical point is the strain dependency of the phenotype (Shinagawa and Kojima 2003). In order to overcome these limitations, other species have been used to establish models of allergic bronchial asthma, including rodents like rats or guinea pigs as well as sheep, horses, cats and monkeys.

#### Rat

While in mice long-term exposure to the allergen for up to 15 and more challenges is required to induce airway remodeling (Brusasco et al. 1999; Wegmann et al. 2005), structural changes of the airway wall have been observed surprisingly earlier in rat models of experimental asthma. In Brown Norway rats (*Rattus norwegicus*) that were systemically sensitized to OVA using alum and *Bordetella pertussis* vaccine as adjuvants, an increased airway smooth muscle mass in intrapulmonary airways was already observed after three OVA aerosol challenges at day 24 (Sapienza et al. 1991). Subsequent studies using the same protocol further showed increased proliferation and decreased apoptosis of both airway smooth muscle and epithelial cells (Ramos-Barbón et al. 2005), goblet cell hyperplasia (Camateros et al. 2007) and enhanced peribronchial vessel count indicating bronchial angiogenesis (Siddiqui et al. 2013). Furthermore, excessive deposition of collagen type I fibers, bigylcans (versican, biglycan, decorin and lumican) and glycosaminogylcans (chondroitin-, dermatan-, heparin- and keratin sulfate) indicate massive reorganization and remodeling of the extracellular matrix (Pini et al. 2006; Venkatesan et al. 2012). Interestingly, rats that were systemically sensitized against OVA by using alum only required at least four to six OVA aerosol challenges to mimic these signs of airway remodeling (Moir et al. 2003; Leung et al. 2005; Tigani et al. 2007), indicating that the inflammatory and remodeling processes are not necessarily independent from the sensitization protocol.

#### Guinea pig

Models of allergic bronchial asthma in guinea pigs are quite more variable. Prado and colleagues sensitized and challenged these animals by eight OVA aerosol inhalations over a time period of 31 days and reported allergic airway inflammation associated with increased collagen fiber deposition (Prado et al. 2005). Subsequent studies also found enhanced expression and content of elastic fibers and actin in the airway wall as well as thickening of the airway epithelium and the smooth muscle layer (Nakashima et al. 2008; Olivo et al. 2012; Pigati et al. 2015). However, the same protocol also resulted in increased matrix fiber deposition in alveolar walls, which is not a feature a human bronchial asthma (Possa et al. 2012). Guinea pigs have also been sensitized systemically to OVA by using alum as an adjuvant and were subsequently challenged with OVA aerosol for up to 12 weeks, which resembles protocols from mouse models of chronic experimental asthma. Such a protocol results in profound allergic airway inflammation associated with goblet cell hyperplasia, mucus gland hypertrophy, smooth muscle layer thickening and increased collagen deposition (Dekkers et al. 2010; Maarsingh et al. 2011; Lucarini et al. 2014).

#### Sheep

Modeling asthma in non-rodent animals is more extensive due to issues of animal housing under laboratory conditions and to their behavior and socialization as well as simply to their size. However, lung and airway anatomy of sheep is much similar to human anatomy than the lung and airway anatomy in mice, rats and guinea pigs. Since airway remodeling proverbially alters the micro-anatomy of the lung, this is a considerable argument. Arguing that innervation and blood supply of the sheep lung is nearer to the human organ situation than that of mice, Snibson et al. (2005) used subcutaneous injection of HDM and alum to sensitize sheep and exposed these animals overnight to HDM aerosol for 6 months. This protocol produced allergic airway inflammation and airway remodeling by points of goblet cell and epithelial cell hyperplasia as well as of collagen and smooth muscle actin content of the airways. A subsequent study also showed increased airway vessel density, which suggests vascular remodeling (Van Der Velden et al. 2013).

#### Cat

Particularly interesting animals for modeling human bronchial asthma are species that spontaneously develop idiopathic forms of asthma such as cat (*Felis silvestris catus*) and horse (*Equus ferus caballus*). Thus, approximately 1% of the feline population develops a disease characterized by episodes of coughing, mucus hypersecretion and broncho-obstruction and displays AHR as well as airway eosinophilia (Padrid 2000). Kirschvink et al. (2007a, b) sensitized cats to *Ascaris suum* (AS) allergen and induced allergic airway inflammation by AS for aerosol inhalations. The authors found increased activity of matrix-metalloprotease (MMP) 9 and a significant age-related increase in interstitial and total radiographic score as assessed by radiography suggesting ongoing airway remodeling processes.

#### Horse

Horses naturally develop a chronic obstructive respiratory condition termed “heaves”, characterized by AHR, recurrent broncho-obstruction and airway inflammation with production of Th2-type cytokines and, thus, share several similarities with human bronchial asthma (Robinson et al. 2000). Animals suffering from the mild-to-moderate type of heaves, which is also termed “inflammatory airway disease (IAD)”, display airway inflammation with neutrophils and eosinophils, while during clinical exacerbation of heaves neutrophils become the dominating leukocyte subpopulation in airway infiltrates (Jean et al. 2011). Herszberg et al. (2006) exposed naturally hay-sensitized horses to barn-dust for 30 consecutive days and recorded increased mucus production and mucus plugging of the airway together with signs of smooth muscle cell hyperplasia by points of increased α-smooth muscle actin mass and enhanced proliferation and reduced apoptosis of smooth muscle cells. A follow-up study also revealed increased collagen content of the airway lamina propria, which positively correlated with increased airway resistance (Setlakwe et al. 2014).

#### Rhesus monkey

In contrast to the aforementioned species, models using primates such as the rhesus monkey (*Macaca mulata*) offer the advantage that monoclonal antibodies against human gene products display considerable cross-reactivity with primate antigens. Furthermore, one can argue that lung anatomy and physiology of the rhesus monkey is even closer to humans then that of quadrupeds like cats, dogs, sheep and horses. Thus, rhesus monkeys have also been utilized to establish models of allergic bronchial asthma and represent an important part of pre-clinical drug testing. The group of Plopper and Hyde at UC Davis sensitized rhesus monkeys to HDM allergen by subcutaneous and intra-muscular injections with alum and *Bordetella pertussis* as adjuvants followed by two intra-nasal applications of HDM solutions and exposure to HDM aerosol for 11 weeks with three exposures a week. Besides allergic airway inflammation, Th2-type cytokine production and development of AHR, this protocol also resulted in airway remodeling with goblet cell hyperplasia, increased thickening of the basement membrane zone, smooth muscle hypertrophy, enhanced subepithelial vascular density and VEGF expression in the airways as well as elevated density of epithelial nerves (Evans et al. 2002; Larson et al. 2004; Tran et al. 2004; Avdalovic et al. 2006). Moreover, this variety of structural changes were demonstrated by using design-based stereology approaches on clearly identified airway generations (for review, see Plopper et al. 2007). Most notably, tracheal RBM thickening persisted after 6-month recovery from allergen exposure (Evans et al. 2004), indicating that, according to our definition, pathological airway remodeling is induced in this model.

### Other models

Without doubt, such asthma models in non-rodent animals offer the advantage of an anatomy and physiology of the lung that in a number of aspects more closely mimics the human situation. Further, in animals larger than rodents, various parameters to assess the asthmatic phenotype can be recorded without sacrificing the study object. However, the question as to which additional insights into the mechanisms of airway remodeling can be obtained by using these model organisms is in general hard to answer. Especially for cats, sheep and horses, reagents for intervention and genetically manipulated strains are only rarely available, if at all. On the contrary, one might argue that, instead of using more and more complex non-rodent animal models that are expensive, time-consuming and pose high demands on ethical standards, the opposite strategy, i.e., using less complex model systems, offers some advantages.

#### Fruit fly

For several years, we have been using the fruit fly *Drosophila melanogaster* as a non-vertebrate model system in respiratory research in order to study innate immunity and remodeling processes of the airway epithelium (Roeder et al. 2009). At a first glance, it might appear all but straightforward to use an animal that lacks active breathing for studying a pulmonary disorder. However, even though the respiratory system of the fruit fly is only an analogue organ to the human lung, there are a few but important similarities between these two systems that recommend using this model system to investigate distinct processes of respiratory biology such as epithelial remodeling of the airways.

**Fig. 3:**
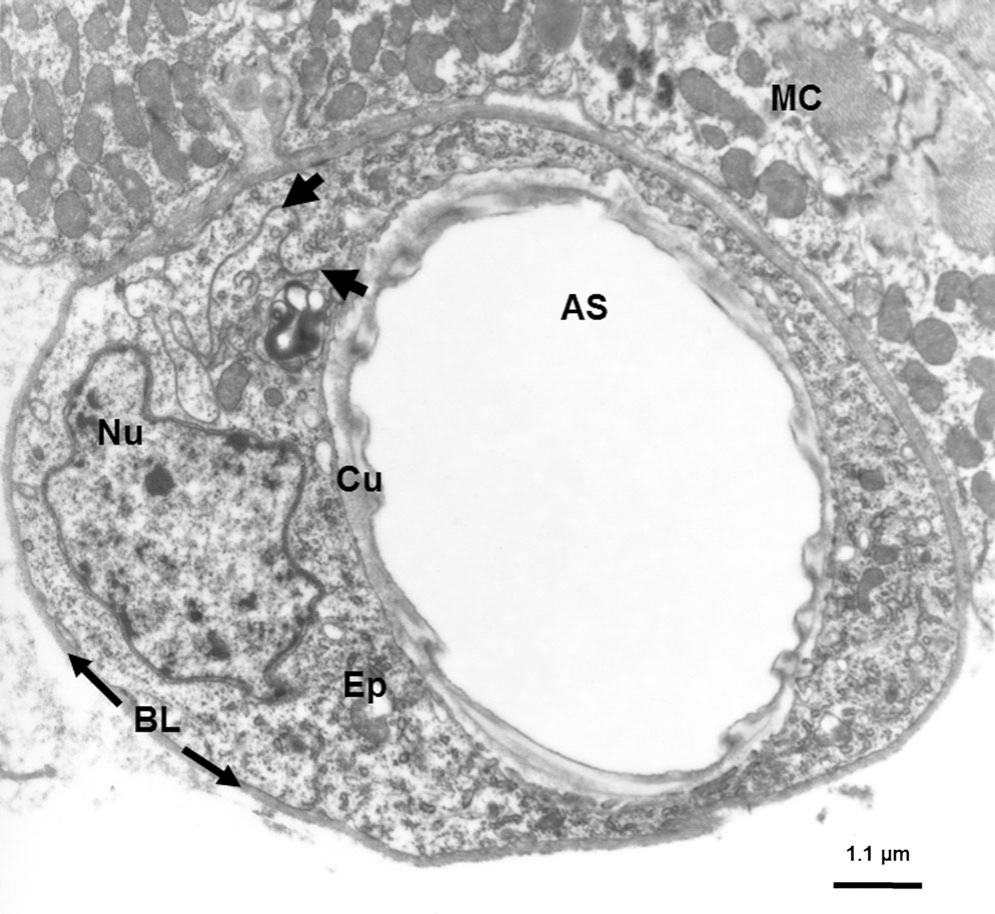
Transmission electron micrograph of a cross-section through a terminal airway branch derived from the respiratory tract of *Drosophila melanogaster*, 3rd instar larva. Tissue was processed as described elsewhere (Fehrenbach et al. 1987). Ultrathin sections were cut on an Ultracut E microtome, collected on formvar-coated nickel grids, stained with lead citrate and analyzed using a Zeiss EM 900. *AS* airway space; *BL* basal lamina; *Cu* cuticula; *Ep* epithelium; *Nu* nucleus and *MC* muscle cells. *Black arrowheads* indicate a cellular junction

In humans as well as in flies, respiration is enabled by the airways that form a tubular network repeatedly branching from proximal to distal, where they end blindly (Rühle 1932). In both species, epithelial cells constitute the barrier between the organism and the environment, thereby mediating the gas exchange. While in humans a variety of different cell types and tissues constitute the airways, the airways of the fruit fly, termed trachea to tracheoli (Fig. 3), are formed by a single layered and entirely immune-reactive epithelium (Wagner et al. 2008). Its innate immune system shares a lot of genes and molecular pathways (e.g., TNF-alpha signaling) with the human one, which is particularly true at the level of recognition, signaling and effectuation (Wagner et al. 2008). This strictly pure epithelial character of the airways combined with the lack of an adaptive immune system makes the fly a highly interesting model system that can be used to exclusively study epithelial remodeling without having to take into account the impact of immune cells like T cells, B cells, mast cells and eosinophils. The key role of the airway epithelium in asthma pathogenesis being widely recognized (Holgate 2007; Fahy and Locksley 2011; Lambrecht and Hammad 2014), the unique features of *Drosophila* airways allow the use of this model system primarily to investigate molecular aspects of airway remodeling.

So far, *Drosophila* has been exclusively utilized to elucidate highly conserved signaling pathways, which have been implicated in the pathophysiology of asthma. In this context, the focus was on NF-ĸB signaling using ectopic activation of the NF-ĸB signaling pathway immune deficiency (Imd), which is the most crucial signaling pathway in mediating epithelial immune responses in the fruit fly (Tzou et al. 2000; Wagner et al. 2008). A detailed morphological study of these transgenic airways revealed that permanent Imd activation results in epithelial proliferation and thickening throughout the entire airway tree, indicating metaplastic transformation (Wagner and co-workers, unpublished). Due to this increased thickness, the regular structure and arrangement of the epithelial cells were lost. The airway lumen appeared irregular and compressed, suggesting that the animals were not sufficiently supplied with oxygen any longer. From points of metaplastic transformation of the airway epithelium, this phenotype largely resembles the murine phenotype observed when using ectopic activation of NF-ĸB signaling in mice (Pantano et al. 2008).

The major advantage of the fly is that its genome can be manipulated rapidly and easily due to the availability of a variety of genetic tools, which are freely accessible for anyone. Genes can be selectively switched on or off (overexpression, RNAi) in the airway epithelium by crossing a fly line tagging the airway epithelium specifically to a line carrying the gene of interest. Thus, this simple non-vertebrate model exhibits a very promising potential to decipher, e.g., the functional relevance of novel candidate genes suggested to be implicated in airway epithelial remodeling.

## Outlook

Evidence has increased that asthma can no longer be considered as a single disease entity but rather as a syndrome comprising several pheno-/endotypes, which are characterized by distinct underlying molecular mechanisms. Whether distinct features of airway remodeling are linked specifically to certain pheno-/endotypes awaits future investigation. Hopefully, such studies will implement design-based stereology, which has challenged some traditional views deduced from assumption-based methods, for a reliable assessment of airway remodeling. A plethora of data was collected from both human and animal studies supporting the notion that airway remodeling can be the result of an inflammatory process. However, more and more studies suggest that airway remodeling may alternatively evolve as a primary event initiated very early in life in the absence of any symptoms or of any detectable inflammation. With the advancement of novel in vivo imaging techniques, monitoring of the development and growth of the airways as well as of airway inflammation will be feasible so that our present knowledge on the initiation of airway remodeling can be expected to be significantly expanded. The broad range of animal models presently available from invertebrate to primate species shall be considered as an extraordinary armamentarium giving us the opportunity to select the appropriate model in the context of the specific question to be answered rather than trying to identify “the one-and-only asthma model”, which in an asthma pheno-/endotype world appears to be quite out-dated.

## Footnotes

1 Epithelial secretory products such as mucus and/or surfactant are, however, components that separate the air from the epithelial tissue layer.
